# Manipulating the revision of reward value during the intertrial interval increases sign tracking and dopamine release

**DOI:** 10.1371/journal.pbio.2004015

**Published:** 2018-09-26

**Authors:** Brian Lee, Ronny N. Gentry, Gregory B. Bissonette, Rae J. Herman, John J. Mallon, Daniel W. Bryden, Donna J. Calu, Geoffrey Schoenbaum, Etienne Coutureau, Alain R. Marchand, Mehdi Khamassi, Matthew R. Roesch

**Affiliations:** 1 Department of Psychology, University of Maryland, College Park, Maryland, United States of America; 2 Neuroscience and Cognitive Science Program, University of Maryland, College Park, Maryland, United States of America; 3 Department of Anatomy and Neurobiology, University of Maryland, Baltimore, Maryland, United States of America; 4 NIDA Intramural Research Program, Baltimore, Maryland, United States of America; 5 CNRS, Institut de Neurosciences Cognitives et Intégratives d'Aquitaine (INCIA, UMR 5287), Bordeaux, France; 6 Université de Bordeaux, INCIA, Bordeaux, France; 7 Institute of Intelligent Systems and Robotics, Sorbonne Université, CNRS, Paris, France; University of Oxford, United Kingdom of Great Britain and Northern Ireland

## Abstract

Recent computational models of sign tracking (ST) and goal tracking (GT) have accounted for observations that dopamine (DA) is not necessary for all forms of learning and have provided a set of predictions to further their validity. Among these, a central prediction is that manipulating the intertrial interval (ITI) during autoshaping should change the relative ST-GT proportion as well as DA phasic responses. Here, we tested these predictions and found that lengthening the ITI increased ST, i.e., behavioral engagement with conditioned stimuli (CS) and cue-induced phasic DA release. Importantly, DA release was also present at the time of reward delivery, even after learning, and DA release was correlated with time spent in the food cup during the ITI. During conditioning with shorter ITIs, GT was prominent (i.e., engagement with food cup), and DA release responded to the CS while being absent at the time of reward delivery after learning. Hence, shorter ITIs restored the classical DA reward prediction error (RPE) pattern. These results validate the computational hypotheses, opening new perspectives on the understanding of individual differences in Pavlovian conditioning and DA signaling.

## Author summary

In classical or Pavlovian conditioning, subjects learn to associate a previously neutral stimulus (called “conditioned” stimulus; for example, a bell) with a biologically potent stimulus (called “unconditioned” stimulus; for example, a food reward). In some animals, the incentive salience of the conditioned stimuli is so strong that the conditioned response is to engage the conditioned stimuli instead of immediately approaching the food cup, where the predicted food will be delivered. These animals are referred to as “sign trackers.” Other animals, referred to as “goal trackers,” proceed directly to the food cup upon presentation of the conditioned stimulus to obtain reward. Understanding the mechanisms by which these divergent behaviors develop under identical environmental conditions will provide powerful insight into the neurobiological substrates underlying learning. Here, we test predictions made by a recent computational model that accounts for a large set of studies examining goal-/sign-tracking behavior and the role that dopamine plays in learning. We show that increasing the duration of the time between trials leads more to the development of a sign-tracking response and to the release of dopamine in the nucleus accumbens. During conditioning with shorter intertrial intervals, goal tracking was more prominent, and dopamine was released upon presentation of the conditioned stimulus but not during the time of reward delivery after training. Thus, shorter intertrial intervals restored the classical dopamine reward prediction error pattern. Our results validate the computational hypothesis and open the door for understanding individual differences to classical conditioning.

## Introduction

Lesaint and colleagues [1] recently proposed a new computational model—the “STGT model” (for sign tracking and goal tracking)—which accounts for a large set of behavioral, physiological, and pharmacological data obtained from studies investigating individual variation in Pavlovian conditioned approach behavior [2–8]. Most notably, the model can account for recent work by Flagel and colleagues (2011) that has shown that phasic dopamine (DA) release does not always correspond to a reward prediction error (RPE) signal arising from a classical model-free (MF) system [9].

In their experiments, Flagel and colleagues trained rats on a classical autoshaping procedure, in which the presentation of a retractable-lever conditioned stimulus (CS; 8 s) was followed immediately by delivery of a food pellet (unconditioned stimulus [US]) into an adjacent food cup. In procedures like this, some rats, known as sign trackers (STs), learn to rapidly approach and engage the CS lever, whereas other rats, known as goal trackers (GTs), learn to approach and enter the food cup upon presentation of the CS lever. Although both sign and goal trackers learn the CS-US relationship equally well, it was elegantly shown that phasic DA release in the nucleus accumbens core (NAc) matched RPE signals only in STs [4]. Specifically, during learning in ST rats, DA release to reward decreased, while DA release to the CS increased. In contrast, even though GTs acquired a Pavlovian conditioned approach response, DA release to reward did not decline, and CS-evoked DA was weaker. Furthermore, administration of a DA antagonist blocked acquisition of the ST conditioned response but did not impact the GT conditioned response [4,10].

Several computational propositions have argued that these data could be interpreted in terms of different contributions of model-based (MB)—with an explicit internal model of the consequences of actions in the task—and MF—without any internal model—reinforcement learning (RL) in GTs and STs during conditioning [1,11]. Nevertheless, only the STGT model predicted that manipulating the intertrial interval (ITI) should change DA signaling in these animals: the model suggests that GTs revise the food cup value multiple times during and in between trials during the 90-s ITI. During the trial, the food cup gains value because reward is delivered; however, visits to the food cup during the ITI do not produce reward, thus reducing the value assigned to the food cup. This mechanism prevents the progressive transfer of reward value signal in the model from US time to CS time and hence explains the absence of DA RPE pattern in goal trackers. This aspect of the model predicts that decreasing the ITI should reduce the amplitude of US DA burst (i.e., less time to negatively revise the value of the food cup and reduce the size of the RPE) and that higher food cup value should lead to an increase in the tendency to GT in the overall population. In contrast, increasing the ITI should have the opposite effect. That is, lengthening the ITI and therefore increasing the number of nonrewarded food cup entries should increase the amplitude of US DA burst (i.e., more time to negatively revise the value of the food cup during the ITI and increase the size of the RPE) and lower the value of the food cup, leading to a decreased tendency to GT and an increase tendency to ST. The latter would be accompanied by a large phasic DA response to the highly salient lever CS, as previously observed in STs [4]. Here, we tested these predictions by recording DA release in the NAc using fast-scan cyclic voltammetry (FSCV) during 10 d of Pavlovian conditioning in rats that had either a short ITI of 60 s or a long ITI of 120 s.

## Results

### Longer ITIs increased sign tracking and DA release to the CS and US

DA release was recorded from NAc (S1B–S1E Fig) during a standard Pavlovian conditioned approach behavior task (S1A Fig) for 10 d. Each trial began with the presentation of a lever (CS) located to the left or right side of a food cup (counterbalanced) for 8 s. Upon the lever’s retraction, a 45-mg sucrose pellet was delivered into the food cup, independent of any interaction with the lever. Each behavioral session consisted of 25 trials presented at a random time interval of either 60 s (*n* = 7 rats) or 120 s (*n* = 12 rats). To quantify the degree to which rats engaged in sign- versus goal-tracking behavior, we used the Pavlovian Conditioned Approach (PCA) index [12], which comprised the average of three ratios: (1) the response bias, which is (Lever Presses − Food Cup Entries) / (Lever Presses + Food Cup Entries), (2) the probability (P) difference, which is (P_lever_ − P_receptacle_), and (3) the latency index, which is ($\bar{x}$ Cup Entry Latency − $\bar{x}$ Lever Press Latency) / 8. All of these ratios range from −1.0 to +1.0 (similarly for PCA index) and are more positive and negative for animals that sign track and goal track, respectively. All behavioral indices were derived from sessions during which DA was recorded. For the initial analysis described in this section, behavior and DA were examined across all sessions; the development of behavior and DA over training is examined in later sections.

**Fig 1:**
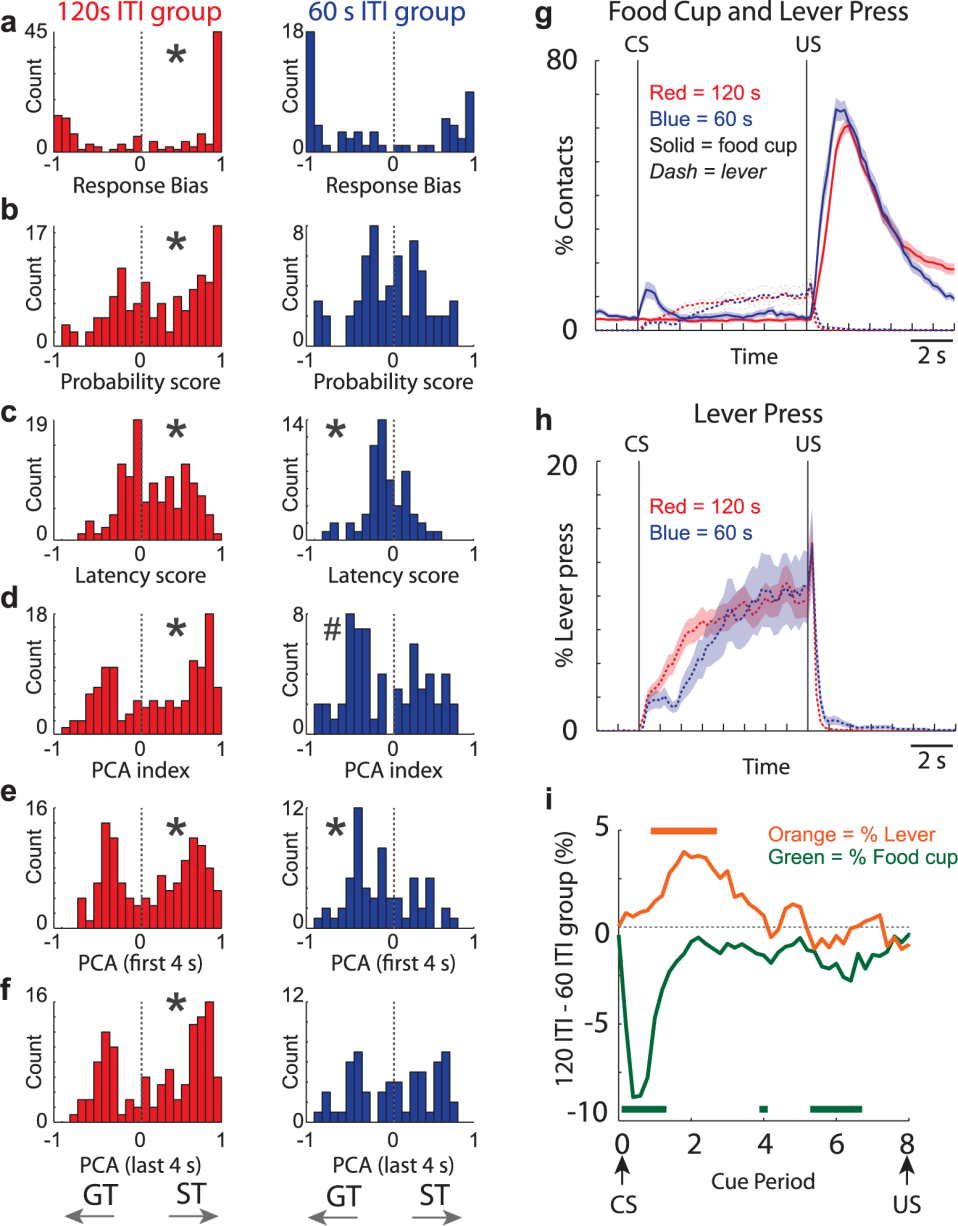
Sign tracking is more prominent during sessions with 120-s ITIs. **(A)** Response bias, which is (Lever Presses − Food Cup Entries) / (Lever Presses + Food Cup Entries). **(B)** The probability difference, which is (P_lever_ − P_receptacle_). **(C)** Latency index, which is ($\bar{x}$ Cup Entry Latency − $\bar{x}$ Lever Press Latency) / 8. **(D)** PCA index = average of response bias, probability difference, and latency difference indices described in A–C. All of these ratios range from −1.0 to +1.0 and are more positive and negative for animals that sign track and goal track, respectively. All behavioral indices are derived from sessions during which DA was recorded (60-s ITI groups = 7 rats; 120-s ITI group = 12 rats) and used behavior during the entire 8-s CS epoch. Each of the above distributions was computed by session. Supporting material reports results by rat. **(E-F)** PCA index computed using just the first 4 s (E) and last 4 s (F) of the CS period;120-s ITI group = red; 60-s ITI group = blue. **(G)** Average beam break (solid) and lever press (dashed) rate for 120-s (red) and 60-s (blue) ITI sessions. **(H)** Average lever press rate for 120-s (red) and 60-s (blue) ITI sessions. Data are the same as in G but with a smaller scale so that differences and timing can be better visualized. Error bars represent SEM. **(I)** Green lines are the difference between solid blue and solid red lines from “G” (food cup entries for the 120-s ITI group minus food cup entries for the 60-s ITI group) during the cue period. Thus, negative deflections illustrate more food cup entries during sessions with a 60-s ITI. Orange lines represent the differences between the 120-s ITI group lever pressing and the 60-s ITI group lever pressing (i.e., red dashed minus blue dashed from “H”). Thus, positive deflections represent time during the cue period when rats in the 120-s ITI group lever pressed more than those in the 60-s ITI group. Orange and green tick marks represent 100-ms bins in which there was a significant difference between 120-s and 60-s ITI groups (*t* test; *p* < 0.05). Percentages for each session were computed and then averaged across sessions. Underlying data for Fig 1 can be found in S1 Data. For analysis averaged within session and rat, and then averaged across rats, please see S2 Fig. CS, conditioned stimulus; DA, dopamine; GT, goal tracking; ITI, intertrial interval; P_lever_, probability of pressing lever; P_receptacle_, probability of entering the receptacle; PCA, Pavlovian Conditioned Approach; ST, sign tracking; US, unconditioned stimulus.

The distributions of behavioral session scores are shown in Fig 1A–1D for each group. As predicted, rats with the 120-s ITI tended to sign track more, whereas rats with the 60-s ITI tended to goal track more. Across all behavioral indices (i.e., response bias, probability, latency, PCA), the mean distributions were positive (biased toward sign tracking) and significant from sessions for rats in the 120-s ITI group (Fig 1A–1D, left; Wilcoxon; μ’s > 0.17, *p* < 0.05). Opposite trends were observed in the 60-s ITI group in that all distributions were negatively shifted from zero (Fig 1A–1D, right; Wilcoxon; response bias: μ = −0.06, *p* = 0.06; lever probability: μ = −0.03, *p* = 0.58; PCA index: μ = −0.11, *p* = 0.097); however, only the shift in the latency difference distribution reached significance (Fig 1C, right; Wilcoxon; μ = −0.10; *p* < 0.05). Direct comparisons between 60-s and 120-s ITI groups produced significant differences across all four measures (Wilcoxons; *p* < 0.01). Thus, we conclude that lengthening the ITI increased sign-tracking behavior, as predicted by the STGT model [1,13].

Notably, the degree of sign/goal tracking within the 60-s ITI group was highly dependent on when behavior was examined during the 8-s CS period. This is illustrated in Fig 1G and Fig 1H, which show percent beam breaks in the food cup (solid lines) and lever pressing (dashed lines) over the time of the trial. Consistent with the ratio analysis described above (Fig 1A–1D), rats in the 120-s ITI group (red) showed sustained pressing (red dashed) that started shortly after lever extension and persisted throughout the 8-s CS period, while showing no increase in food cup entries (red solid) after CS presentation (Fig 1G, red solid versus dashed).

Although it is clear that rats in the 120-s ITI group sign track more than goal track during the CS period, the relationship between lever pressing and food cup entry was far more dynamic during sessions with 60-s ITIs (Fig 1G; blue). During 60-s ITI sessions, rats would briefly enter the food cup for approximately 2 s immediately upon CS presentation (Fig 1G, solid blue), before engaging with the lever (Fig 1G, dashed blue). As a result, lever pressing was delayed in the 60-s ITI group relative to the 120-s ITI group (Fig 1G and 1H; blue versus red dashed). This suggests that the goal-tracking tendencies described above during the entire 8-s CS period were largely due to the distribution of behaviors observed early in the CS period. To quantify this observation, we recomputed the PCA index using either the first or the last 4 s of the 8-s CS period. For the 120-s ITI group, the PCA index was significantly shifted in the positive direction during both the first and last 4 s of the cue period (i.e., more sign tracking; Fig 1E and 1F, left; Wilcoxon; μ’s > 0.16; *p* < 0.05). For the 60-s ITI group, the PCA index was significantly shifted in the negative direction during the first 4 s (i.e., more goal tracking; Fig 1E, right; Wilcoxon; μ = −0.16; *p* < 0.05) but not significantly shifted during the last 4 s (Fig 1F, Wilcoxon; μ = 0.01; *p* = 0.81). Interestingly, this part of the results goes beyond the STGT model, which simplifies time by considering a single behavior/action during that period.

To further demonstrate sign- and goal-tracking tendencies over the 8-s cue period and the differences between groups, we simply subtracted 60-s ITI lever pressing and food cup entries from 120-s ITI lever pressing (Fig 1I; orange) and food cup entries (Fig 1I; green), respectively. Shortly after cue onset, the green line representing the difference between 120-s and 60-s ITI food cup entries dropped significantly below zero. Throughout the cue period (8 s), there were more contacts with the food cup in sessions with a 60-s ITI compared with the 120-s ITI group (green tick marks represent differences between 120-s and 60-s ITI across sliding 100-ms bins; *t* test; *p* < 0.05). For lever pressing (orange), values were constantly higher shortly after the cue for the first half of the cue period (orange tick marks represent differences between 120-s and 60-s ITI across sliding 100-ms bins; *t* test; *p* < 0.05), indicating that there were more contacts with the lever in sessions with a 120-s ITI compared with the 60-s ITI group early in the cue period.

The behavioral data described above globally support model predictions that increasing and decreasing the ITI would produce more and less sign tracking, respectively. Nevertheless, they also pave the way for improvements of the model by showing a rich temporal dynamic of behavior during the trial, rather than the single behavioral response per trial simulated in the model. By plotting lever presses and food cup entries over time, we see that sometimes rats initially go to the lever and then go to the food cup, or vice versa. In contrast, the model was designed to account only for the initial action performed by rats. This was sufficient to account for the main results of the present study. Nevertheless, it would be interesting to extend the model to enable it to account for different decisions made sequentially by the same animal during a given trial.

**Fig 2:**
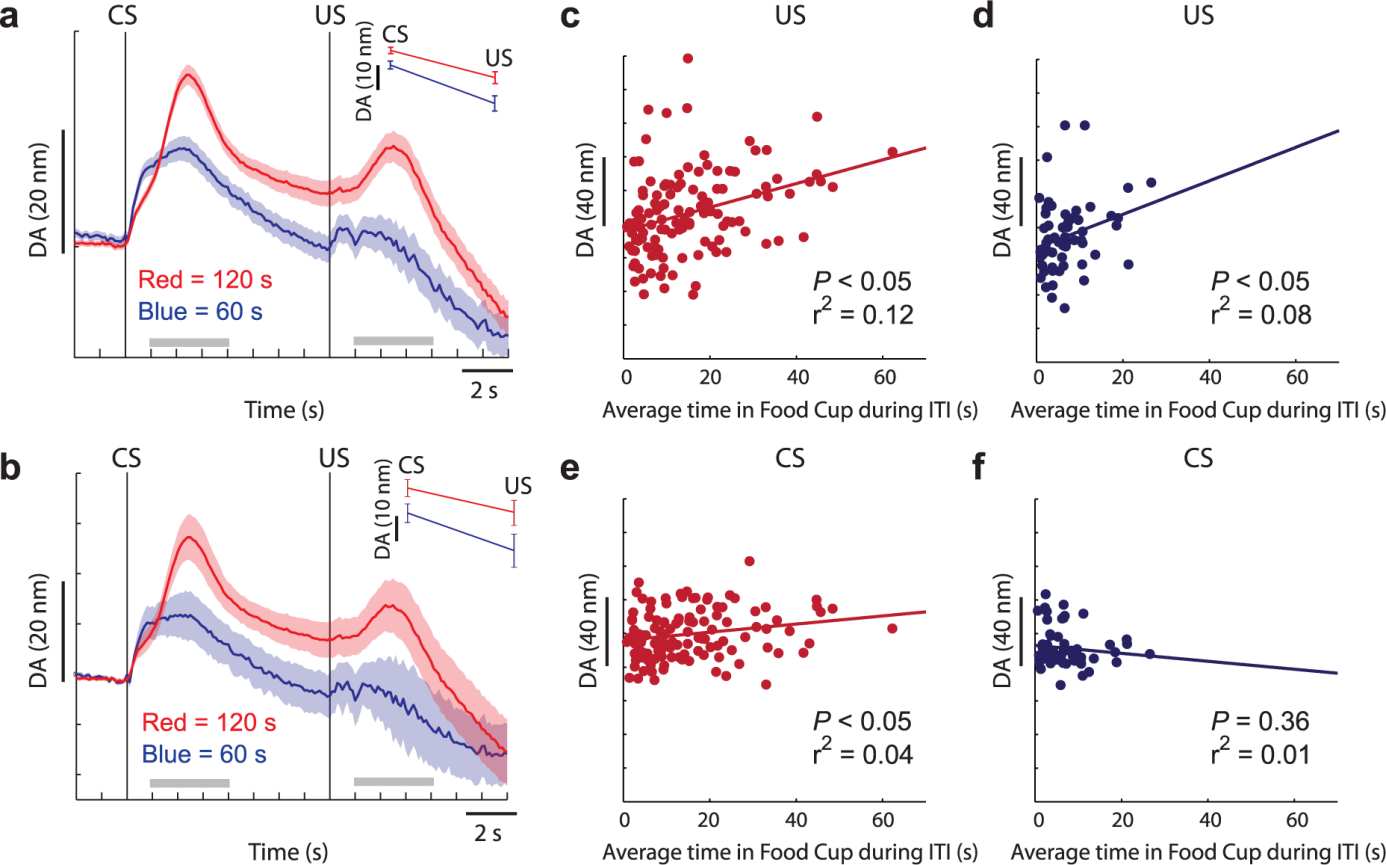
DA release to the CS and US is stronger during sessions with 120-s ITIs. **(A-B)** Average DA release over time for 120-s (red) and 60-s (blue) ITI groups sessions. “A” represents DA release averaged within the session first and then across sessions, whereas DA release in “B” represents averages taken within each session and rat, and then averaged across rats. Error bars represent SEM, with “*n*” being session and rat for “A” and “B,” respectively. **(C-D)** Average DA release during the US period (y-axis) relative to time the photobeam in the food cup was broken during the ITI for the 120-s (red) and 60-s (blue) ITI groups for each session. **(E-F)** Average DA release during the CS period (y-axis) relative to time the photobeam in the food cup was broken during the ITI for the 120-s (red) and 60-s (blue) ITI groups. Underlying data for Fig 2 can be found in S2 Data. CS, conditioned stimulus; DA, dopamine; ITI, intertrial interval; US, unconditioned stimulus.

Next, we tested the prediction that longer ITIs would elevate DA release to the US, while shorter ITIs would reduce DA release to the US. The average DA release over all sessions for the 60-s and 120-s groups is shown in Fig 2A. Rats in the 120-s ITI group exhibited significantly higher DA release to the CS and the US relative to rats in the 60-s ITI group (CS *t* test: *t* = 2.99, df = 178, *p* < 0.05; US *t* test: *t* = 3.07, df = 178, *p* < 0.05). In the 120-s ITI group, DA release to both the CS and the US was significantly higher than baseline (CS *t* test: *t* = 14.77, df = 119, *p* < 0.05; US *t* test: *t* = 4.79, df = 119, *p* < 0.05); however, in the 60-s ITI group, this was only true during CS presentation (*t* test: *t* = 7.34, df = 59, *p* < 0.05); DA release at the US was not different than baseline (*t* test: *t* = 0.99, df = 59, *p* = 0.33). Similar results were obtained when averaging across sessions within each rat and then averaging across rats (Fig 2B); DA release was higher during the CS and US for rats in the 120-s ITI group (CS *t* test: *t* = 1.87, df = 17, *p* < 0.05; US *t* test: *t* = 1.83, df = 17, *p* < 0.05) and was higher than baseline for both periods (CS *t* test: *t* = 6.15, df = 11, *p* < 0.05; US *t* test: *t* = 2.16, df = 11, *p* < 0.05), whereas DA release was only significantly higher during the CS period for rats in the 60-s ITI group (CS *t* test: *t* = 6.68, df = 6, *p* < 0.05; US *t* test: *t* = 0.70, df = 6, *p* = 0.26).

These results are in line with the STGT model, which predicted that reducing ITI duration would prevent the downward revision of the food cup value and hence would permit the high predictive value associated with the food cup to produce a DA response at CS but not US, consistent with the DA RPE hypothesis [9]. Conversely and also consistent with model predictions, DA release during sessions with the longer ITI was significantly higher during US delivery because there were more positive RPEs, which may result from the positive surprise associated with being rewarded in a food cup whose value has been more strongly decreased during multiple visits to the food cup during long ITIs. Nevertheless, at the CS time, the increased DA burst at CS indicates an even more complex process that goes beyond model predictions.

All of this suggests that DA release should be positively correlated with the time spent breaking the beam in the food cup during the ITI. To test this hypothesis, we computed how much time was spent in the food cup during the ITI for each session. This was done by determining the total number of beam breaks within each ITI (10-ms resolution) and then averaging over trials to determine each session mean. Importantly, the ITI time did not vary across sessions within each group, and the analysis was performed separately for the two groups (60-s group and 120-s group). Thus, any correlation between DA and food cup interaction time during the ITI cannot reflect a correlation between DA and ITI time. As expected, rats in the 120-s ITI group spent significantly more time in the food cup than did rats in the 60-s ITI group (120-s ITI group = 15.1 s; 60-s ITI group = 6.8 s; *t* test: *t* = 4.91, df = 178, *p* < 0.05). For both groups, there was a significant positive correlation between average time spent in the food cup during the ITI and DA release during the reward period (Fig 2C, 120-s ITI: r^2^ = 0.12, *p* < 0.05; Fig 2D, 60-s ITI: r^2^ = 0.08, *p* < 0.05). During the cue period for the 120-s ITI group, but not the 60-s ITI group, there was also positive correlation (Fig 2E, 120-s ITI: r^2^ = 0.04, *p* < 0.05; Fig 2F, 60-s ITI: r^2^ = 0.01, *p* = 0.36). Finally, when examining with data collapsed across both groups, there was a significant positive correlation during both cue and reward epochs (Cue: r^2^ = 0.05, *p* < 0.05; Reward: r^2^ = 0.14, *p* < 0.05). Thus, we conclude that DA release to the CS and US tended to be higher the longer rats visited the food cup during the ITI.

### Development of sign tracking and DA signals over training

In the analysis above, we averaged DA release and behavior from all recording sessions. Next, we asked how behavior and DA release patterns evolved with training. As a first step to addressing this issue, we recomputed the PCA analysis for the first and last 5 d of training. For the 60-s ITI group, the PCA index distribution was significantly shifted in the negative direction (i.e., goal tracking) during the first five sessions (Wilcoxon; μ = −0.38, *p* < 0.05) but not in the last five sessions (Wilcoxon; μ = 0.15, *p* = 0.07). Thus, early in training, rats with the 60-s ITI exhibited goal tracking more than sign tracking but did not fully transition to sign tracking, at least when we averaged over the last five sessions. For the 120-s ITI group, the PCA index was significantly shifted in the positive direction (i.e., sign tracking) during the last five sessions (Wilcoxon; μ = 0.28, *p* < 0.05) but was not during the first five sessions (Wilcoxon; μ = 0.10, *p* = 0.11). Thus, when the ITI was long (120 s), rats sign and goal tracked in roughly equal proportions during the first five sessions but tended to sign track significantly more during later sessions.

**Fig 3:**
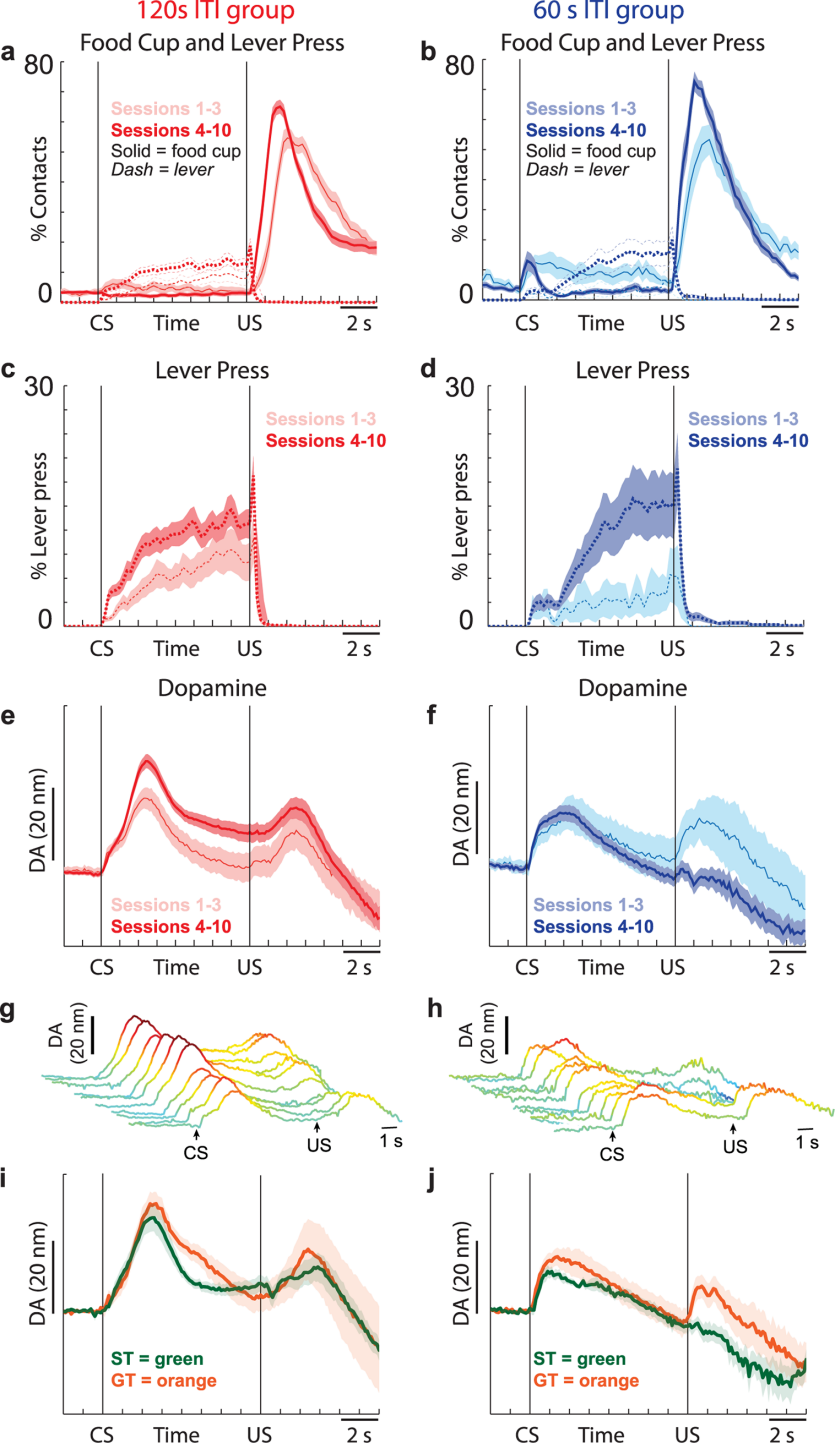
Development of sign tracking and DA signals over training. **(A-B)** Average beam break (solid) and lever press (dashed) rate for 120-s (A) and 60-s (B) ITI sessions. **(C-D)** Average lever press rate for 120-s (C) and 60-s (D) ITI sessions. Percentages for each session were computed and then averaged across sessions. Data are the same as in A and B but with a smaller scale so that differences and timing can be better visualized. **(E-F)** Average DA release over time for 120-s (E) and 60-s (F) ITI sessions. In each of the above (A-F), data are broken down into averages from sessions 1–3 (pale colors, pink [120 s] and turquoise [60s]) and sessions 4–10 (dark colors, red [120 s] and blue [60 s]); 60-s ITI group = 7 rats; 120-s ITI group = 12 rats. DA release for each session was computed and then averaged across sessions. For analysis averaged within session and rat and then averaged across rat, please see S2 Fig. **(G-H)** Average DA release over time for each of the 10 sessions for the 120-s ITI group (G) and the 60-s ITI group (H). For breakdown of behavior by session for each of the 10 sessions, please see S4 Fig. **(I-J)** Average DA release over time for 120-s (I) and 60-s (J) ITI groups broken down into sessions in which rats either lever pressed (120-s ITI = 4 rats, 20 sessions; 60-s ITI = 2 rats, 7 sessions) or entered the food cup during the CS (120-s ITI = 3 rats, 13 sessions; 60-s ITI = 4 rats, 18 sessions). Error bars represent SEM. Underlying data for Fig 3 can be found in S3 Data. CS, conditioned stimulus; DA, dopamine; GT, goal tracking; ITI, intertrial interval; ST, sign tracking; US, unconditioned stimulus.

To more accurately pinpoint when during training rats in the 120-s group shift toward sign tracking, we examined the four distributions individually for each session. Sign tracking became apparent during session 4, when the latency and lever probability distributions first became significant (Wilcoxon; latency: μ = 0.28, *p* < 0.05; lever probability: μ = 0.40, *p* < 0.05). To visualize changes in behavior and DA release that occurred before and after session 4, we plotted food cup beam breaks, lever pressing, and DA release averaged across the first 3 d of training and across days 4–10 (Fig 3; for visualization of behavior during each of the 10 sessions, please see S4 Fig). Consistent with the distributions of behavioral indices described above, the 120-s ITI group showed roughly equal food cup entries and lever pressing during the CS period in the first 3 d of training (Fig 3A, thin pink solid versus thin pink dashed), whereas later in training (days 4–10; red), there was a strong preference for the lever (Fig 3A; thick red dashed versus thick red solid). Indeed, the distribution of PCA indices averaged during days 4–10 were significantly shifted in the positive direction (Wilcoxon; μ = 0.27, *p* < 0.05).

These results suggest that in sessions in which the ITI was set at 120 s, sign-tracking tendencies developed relatively quickly during the first several recording sessions (Fig 3A and 3C). This is consistent with the STGT model, which predicted that increasing the ITI duration would increase the global tendency to sign track within the population and would thus speed up the acquisition of lever pressing behavior [1,13]. In contrast, the model also predicted that reducing the ITI duration would increase the global tendency to goal track and would thus slow down the acquisition of lever pressing behavior. Interestingly, the behavior of the 60-s ITI group was far more complicated than behavior of the 120-s group, with changes in goal and sign tracking occurring over training and CS presentation time. Early in training, rats in the 60-s ITI group clearly visited the food cup (Fig 3B, solid turquoise) more than they pressed the lever (Fig 3B, dashed turquoise); food cup entries increased shortly after presentation of the CS and continued throughout the CS period (Fig 3B, solid turquoise). During later sessions (i.e., 4–10), rats in the 60-s ITI group still entered the food cup upon CS presentation—which corresponds to the goal-tracking behavior predicted by the model in this case—but this only lasted about 2 s, at which point they transitioned to the lever (Fig 3B and 3D). In sessions 4–10, none of the distributions of behavioral indices were significantly shifted from zero when examining the CS period as a whole (Wilcoxons; Response bias: μ = 0.27, *p* = 0.83; Latency: μ = −0.05, *p* = 0.13; Probability: μ = 0.08, *p* = 0.16; PCA: μ = 0.02, *p* = 0.82) or during the first half of the CS period (Response bias: μ = −0.11, *p* = 0.027; Probability: μ = −0.04, *p* = 0.18; PCA: μ = −0.07, *p* = 0.25); however, when examining the last 4 s of the CS period, distributions were significantly shifted in the positive direction (Wilcoxons; Response bias: μ = 0.32, *p* < 0.05; Probability: μ = 0.28, *p* < 0.05; PCA: μ = 0.24, *p* < 0.05). Together, this suggests that rats in the 60-s groups were largely goal tracking early in training and that over the course of training, goal-tracking tendencies did not disappear but became focused to early portions of the CS period, while sign-tracking behavior developed toward the end of the CS period, later in training (Fig 3B and 3D; S4 Fig). Interestingly, these results go again beyond the computational model and suggest that it should be extended to account for within-trial behavioral variations.

Behavioral analyses clearly demonstrate that manipulation of the ITI impacts sign- and goal-tracking behavior and that both groups learned that the CS predicted reward (Fig 3; S4 Fig). Next, we determined how DA patterns changed during training. Fig 3E and 3F illustrate DA release averaged across the first 3 d and days 4–10 of sessions with 120-s and 60-s ITIs, respectively, and DA release for each session is plotted in Fig 3G and 3H. As shown previously, both groups started with modest DA release to both the CS and US during the first session (Fig 3G and 3H; trial 1). For the 120-s ITI group, DA release was significantly higher to CS presentation later (red) compared to earlier (pink) in learning (Fig 3E; *t* test: *t* = 2.51, df = 119, *p* < 0.05). DA release during US delivery did not significantly differ between early and late phases of training (*t* test: *t* = 1.27, df = 119, *p* = 0.21). Hence, similarly to the sign trackers in the original study of Flagel and colleagues (2011), the increase of DA response to the CS is consistent with the RPE hypothesis. The difference is that here, the increase in the time available to down-regulate the value associated with the food cup during the ITI may have resulted in a remaining positive surprise at the time of reward delivery, hence preventing the progressive decrease of response to the US across training, in accordance with the model predictions.

In the 60-s ITI group (Fig 3F and 3H), DA release to the US was initially high during the first 3 d (turquoise) but declined during days 4–10 (blue). Directly comparing DA release during the first 3 d with the remaining days revealed significant differences during the US period (*t* test: *t* = 1.14, df = 59, *p* < 0.05) but not the CS period (*t* test: *t* = 0.08, df = 59, *p* = 0.93). As a consequence, their post-training DA pattern—with a high response to the CS but no response to the US (Fig 3F, blue)—now resembles the traditional RPE pattern (i.e., high CS DA and low US DA after learning). This is a clear demonstration that the DA RPE signal can be observed in goal trackers with a manipulation of the ITI, as predicted by the STGT model.

### DA patterns during sign and goal tracking

In a final analysis, we examined DA patterns during pure sign and goal tracking within each ITI group. For this analysis, we examined only sessions during which either the lever was pressed or the food cup was entered during the cue period. As shown previously [4], phasic DA responses were apparent during both the CS and US during sessions with goal tracking (Fig 3I and 3J, GT = orange). In addition to replicating previous results, the figure also illustrates modulation of the DA pattern in line with model predictions. Specifically, it shows that the DA response to the US was higher in the 120-s group than in the 60-s group during both sign- and goal-tracking sessions (sign-tracking: *t* test, *t* = 3.66, df = 25, *p* < 0.05; goal-tracking: *t* test, *t* = 1.44, df = 29, *p* = 0.16) and that the DA response to the US was significantly lower than the DA response to the CS in GTs of the 60-s group (*t* = 3.87, df = 17, *p* < 0.05), suggesting that even though there is still a DA response to the US, shortening the ITI reduced the US-evoked DA response compared with what has been previously reported [4].

## Discussion

The results reported here support the STGT model’s predictions that manipulating the ITI would impact the proportion of sign-tracking (STs) and goal-tracking (GTs) behaviors as well as DA release. It predicted that shortening the ITI would result in fewer negative revisions of the food cup value and reduce the US DA burst. It also predicted that the resulting higher food cup value would lead to an increase in the tendency to GT across sessions [1,13], which it did. The model also predicted that lengthening the ITI would have the opposite effect. We found that there were significantly more food cup entries during the ITI for the 120-s ITI group and that they showed an increased tendency to sign track. Furthermore, we show that the time spent in the food cup during the ITI was positively correlated with the amplitude of the CS and US DA bursts for the 120-s ITI group, which is consistent with the hypothesis that lengthening the ITI to allow for more time to decrease the value of the food cup would result in stronger positive RPEs during the trial.

Consistent with the model, we claim that increased sign tracking and DA release result from the additional time spent in the food cup during the ITI. Indeed, these were positively correlated. Importantly, this impact of ITI manipulations had not been predicted by other computational models of sign trackers and goal trackers [11,14]. However, several alternative explanations should be considered, which may have also contributed to observed changes in behavior and DA release. For example, it has been shown that rewards delivered after longer delays yield higher DA responses to the US [15–17] and that uncertain reward increases sign tracking [18]. Although the reward was highly predictable in our study (i.e., always delivered 8 s after cue onset), it is possible that uncertainty associated with US delivery impacted behavior and DA release. Notably, it is likely that these factors are intertwined in that manipulating delays and certainty impact the number of visits to the food cup that are not rewarded, thus leading to a negative revision of the food cup, as predicted by the model. Future work that modifies food cup entries without manipulating ITI length and rewards uncertainty is necessary to determine the unique contributions that these factors play in goal-/sign-tracking behavior and associated DA release.

Another explanation for increased sign tracking and DA release in the rats in the 120-s ITI group is the possibility that they learned faster than rats in the 60-s ITI group because of differing ratios between US presentations and the interval between the CS and US in that, the shorter the CS-US interval relative to the ITI, the faster the learning [19]. In the context of our study it is difficult to determine which group learned faster. Although rats in the 120-s ITI group did lever press more often early in training, rats in the 60-s ITI group made more anticipatory food cup entries during the cue period prior to reward delivery. Furthermore, both food cup entries and lever pressing were present in the first behavioral session (S4 Fig). Thus, both groups appear to learn the CS-US relationship at similar speeds, but it is just that the behavior readout of learning differs across groups, making it difficult to determine which group learned the association faster. In our opinion, our results suggest that rats in both groups learned at similar rates, much like sign and goal trackers do; however, future experiments and iterations of the model are necessary to determine what role the US-US/CS-US ratio plays in sign/goal tracking and corresponding DA release.

Standard RL [20] is a widely used normative framework for modelling learning experiments [21,22]. To account for a variety of observations suggesting that multiple valuation processes coexist within the brain, two main classes of models have been proposed: MB and MF models [23,24]. MB systems employ an explicit, although approximate, internal model of the consequences of actions, which makes it possible to evaluate situations by forward inference. Such systems best explain goal-directed behaviors and rapid adaptation to novel or changing environments [25–28]. In contrast, MF systems do not rely on internal models but directly associate stored (cached) values with actions or states based on experience, such that higher valued situations are favored. Such systems best explain habits and persistent behaviors [28–30]. Learning in MF systems relies on a computed reinforcement signal, the RPE (actual minus predicted reward). This signal has been shown to correlate with the phasic response of midbrain DA neurons that increase and decrease firing to unexpected appetitive and aversive events, respectively [9,31].

Recent work by Flagel and colleagues [4] has questioned the validity of classical MF RL methods in Pavlovian conditioning experiments. Their autoshaping procedure reported in that article was nearly identical to the one presented here in that a retractable-lever CS was presented for 8 s, followed immediately by delivery of a food pellet into an adjacent food cup. The only major difference was that the length of the ITI in their study was 90 s. In their study, they showed that in STs, phasic DA release in the NAc matched RPE signaling. That is, the DA burst to reward that was present early in learning transferred to the cue after learning. They also showed that DA transmission was necessary for the acquisition of sign tracking. In contrast, despite the fact that GTs acquired a Pavlovian conditioned approach response, this was not accompanied by the expected RPE-like DA signal, nor was the acquisition of the goal-tracking conditioned response blocked by administration of a DA antagonist (see also Danna and Elmer [10]).

To account for these and other results, Khamassi and colleagues [1] proposed a new computational model—the STGT model—that explains a large set of behavioral, physiological, and pharmacological data obtained from studies on individual variation in Pavlovian conditioned approach [2–8]. Importantly, the model can reproduce previous experimental data by postulating that both MF and MB learning mechanisms occur during behavior, with simulated interindividual variability resulting from a different weight associated with the contribution of each system. The model accounts for the full spectrum of observed behaviors ranging from one extreme—from sign tracking associated with a small contribution of the MB system in the model—to the other—goal tracking associated with a high contribution of the MB system in the model [12]. Above all, by allowing the MF system to learn different values associated with different stimuli, depending on the level of interaction with those stimuli, the model potentially explains why the lever CS and the food cup might acquire different motivational values in different individuals, even when they undergo the same training in the same task [26].

The STGT model explains why the RPE-like dopaminergic response was observed in STs but not GTs—the proposition being that GTs would focus on the reward-predictive value of the food cup, which would have been down-regulated during the ITI. Furthermore, the STGT explains why inactivating DA in the core of the nucleus accumbens or in the entire brain results in blocking specific components and not others. Here, the model proposes that learning in GTs relies more heavily on the DA-independent MB system, and thus DA blockade would not impair learning in these individuals [4,8]. More importantly, the model has led to a series of new experimentally testable predictions that assess and strengthen the proposed computational theory and allow for a better understanding of the DA-dependent and DA-independent mechanisms underlying interindividual differences in learning [1,13].

The key computational mechanism in the model is that both the approach and the consumption-like engagement observed in sign trackers (STs) on the lever and in goal trackers (GTs) on the food cup result from the acquisition of incentive salience by these reward-predicting stimuli. Acquired incentive salience is stimulus specific: stimuli most predictive of reward will be the most “wanted” by the animal. The MF system attributes accumulating salience to the lever or the food cup as a function of the simulated DA phasic signals. In the model simulations, because the food cup is accessible but not rewarding during the ITI, a simulated negative DA error signal occurs each time the animal visits the food cup and does not find a reward. The food cup therefore acquires less incentive salience compared with the lever, which is only presented prior to reward delivery. In simulated STs, behavior is highly subject to incentive salience because of a higher weight attributed to the MF system than to the MB system. As a consequence, they are more attracted by the lever than by the food cup. By contrast, simulated GTs are controlled by the MB system with a higher weight than the MF system. This makes them prefer the food cup, which is the shortest path to reward in the MB system. Moreover, because the food cup has a lower incentive salience, simulated GTs engage with the food cup less than STs do with the lever, as observed experimentally.

The STGT model also led to specific predictions about what would happen if rats had more exposure to the food cup in the absence of reward. The key prediction of this aspect of the model was that increased access to the food cup during the ITI should decrease the incentive salience associated with it and, conversely, increase the strength of engagement with the lever. This, in turn, would increase the relative proportion of ST conditioned responses compared to GT conditioned responses. In addition, the model predicts that DA release to the CS would be higher than DA to the US because of the predictive power of the CS, and DA release to the US would remain high after conditioning because of the persistent positive surprise associated with reward delivery in the food cup. Both of these predictions were confirmed in our current study; in sessions from rats in the 120-s ITI group, the tendency to sign track was more prominent, DA release was significantly higher during CS presentation, and US-evoked DA remained after learning. Conversely, the model also predicted that decreased access to the food cup during the ITI would increase the incentive salience associated with the food cup, resulting in more goal-tracking behavior associated with a DA signal better resembling the classic RPE pattern (i.e., cue but not reward firing after learning). These predictions were partially confirmed in our current study; rats in the 60-s ITI group were more likely to be goal trackers if they were classified based on their initial approach to the food cup in response to the CS [4]. The post-learning DA pattern of the 60-s ITI group showed a high DA response to the CS, but not the US. Taken together, these results validate the STGT model.

However, it is worth noting that the observed behavior of the 60-s ITI group goes beyond the predictions of the STGT model. The 60-s ITI group indeed showed more complex behavior in response to lever CS presentation: an initial food cup approach during the first 2 s after the CS—consistent with goal-tracking behavior—followed by a more ST-like behavioral engagement with the lever (Fig 1G and 1H; Fig 3B and 3D; S4 Fig). The late engagement with the lever is not predicted by the computational model, which only attempts to model the initial behavioral response of the animals to the CS [1]. This is compatible with the way sign trackers and goal trackers were classified based on their initial response to the CS in the original study [4]. This simplification in the model still accounts for a full spectrum of interindividual variability, even animals originally classified in the “intermediate group,” exhibiting both ST and GT behaviors. Nevertheless, the present results highlight that the STGT model should be extended to account for temporal variability of the animal’s behavior within each trial.

## Materials and methods

### Animals

Twenty-nine male Sprague-Dawley rats were obtained from Charles River Labs at 250–275 g (90–120 d old). Animals were individually housed in a temperature- and humidity-controlled environment and kept on a 12-h light/dark cycle (0700–1900 in light); all tests were run during the light phase. Animals had access to water ad libitum and body weight was maintained at 85% of baseline weight by food restriction (15 g standard rat chow was provided daily, in addition to approximately 1 g sucrose pellets during experimental trials).

### Ethics statement

All procedures were performed in concordance with the University of Maryland, College Park Institutional Animal Care and Use Committee (protocol number R-15-34).

### Chronic microelectrode fabrication

Electrodes were constructed according to the methods of Clark and colleagues (2010) [32]. A single carbon fiber (Goodfellow Corporation, Coraopolis, PA) was inserted into a 15-mm cut segment of fused silica (Polymicro Technologies, Phoenix, AZ) while submerged in isopropyl alcohol. One end of the silica tubing was sealed with a two-part epoxy (T-QS12 Epoxy, Super Glue) and left to dry overnight, leaving untouched carbon fiber extending past the seal. The protruding carbon fiber was cut to a length of 150 μm. A silver connector (Newark, Chicago, IL) was secured to the carbon fiber at the opposing end of the silica tubing using silver epoxy (MG Chemicals, British Columbia, Canada) and was allowed to dry. A final coat of two-part epoxy was then applied to the pin connection to provide insulation and structural support for the electrode and was allowed to dry overnight.

### Intracranial surgical procedures

All animals were anesthetized using isoflurane in O_2_ (5% induction, 1% maintenance) and implanted with a chronic voltammetry microelectrode aimed at the NAc core (+1.3 AP, +1.8 ML, −6.6 DV), an ipsilateral bipolar stimulating electrode (Plastics One, Roanoke, VA) in the medial forebrain bundle (−2.8 AP, +1.7 ML, −8.5 DV), and a contralateral Ag/AgCl reference electrode (Sigma-Aldrich, Allentown, PA). The reference electrode and anchoring screws were stabilized using a thin layer of dental cement (Dentsply, York, PA), leaving the holes for the stimulating and recording electrodes unobstructed. The stimulating and recording electrodes were attached to a constant current isolator (A-M Systems, Carlsborg, WA) and voltammetric amplifier, respectively, and lowered to the most dorsal point of the target region (−6.6 DV for the working electrode and −8.5 DV for the stimulating electrode). At this depth, a triangular voltammetric input waveform (−0.4 to +1.3 V versus Ag/AgCl, 400 V/s; Heien and colleagues, 2003) was applied to the recording electrode at 60 Hz for 30 min and then reduced to 10 Hz for the remainder of the surgery. Electrical stimulation (24 biphasic pulses, 60 Hz, 120 μA) was applied to the stimulating electrode in order to evoke DA release, which was monitored at increasing depths by the recording electrode. If neither an evoked change in DA nor a physical response (whisker movement or blinking) was observed, the stimulating electrode was lowered by 0.05 mm until a response was achieved or to a maximum depth of 8.8 mm. The working electrode was then lowered by 0.05 mm until DA release was observed or to a maximum depth of 6.9 mm. Once electrically evoked DA release was detected in the NAc core, a thin layer of dental cement was used to secure the stimulating and recording electrodes in place. A Ginder implant (Ginder Scientific [Nepean, Ontario, Canada]; constructed in house) was connected to the reference, stimulating, and recording electrodes and fully insulated using dental cement, leaving only the screw-top connector exposed, in order to reduce noise and prevent loss of connectivity during behavioral training. Animals then received postoperative care: subcutaneous injection of 5 mL saline containing 0.04 mL carprofen (Rimadyl), topical application of lidocaine cream to the surgical area, and placement on a heating pad until full consciousness was regained. Animals were also given antibiotic treatment of Cephlexin orally twice daily post-surgery for 1 wk to prevent infection of the surgical site. All subjects were allowed a month for full recovery and stabilization of the electrode before experimentation.

### Pavlovian training

All behavioral procedures were conducted in Med Associates test chambers equipped with a food cup and a retractable lever located to the left or right of the food cup (counterbalanced). Head entries into the food cup were time-stamped during disruption of the photobeam located inside the receptacle. Similarly, time stamps were generated during downward deflection of the lever.

Three pretraining sessions were conducted that consisted of the delivery of 25 sucrose pellets, which were randomly delivered on a variable-interval 30 ± 15-s schedule. Following pretraining, rats began Pavlovian training sessions, which consisted of the presentation of the lever (CS) for 8 s, which was immediately followed with delivery of a sucrose pellet upon its retraction. The CS was presented on a random interval of either 60 ± 30 s (i.e., 30, 40, 50, 60, 70, 80, or 90 s) or 120 ± 30 s (i.e., 90, 100, 110, 120, 130, 140, or 150 s), and each Pavlovian session consisted of 25 trials. Pavlovian training continued for 10 sessions, which were accompanied with FSCV recording.

### FSCV

For recordings, animals were connected to a head-mounted voltammetric amplifier (current to voltage converter) and a commutator (Crist Instruments, Hagerstown, MD) mounted above the recording chamber. During each session, an electrical potential was applied to the recording electrode in the same manner as described above (see “Intracranial surgical procedures”). In order to detect changes in dopaminergic concentration over time, the current at its peak oxidation potential was plotted for successive voltammetric scans, and background signal was subtracted. Two PC-based systems, fitted with PCI multifunction data acquisition cards and software written in LabVIEW (National Instruments, Austin, TX), were used for waveform generation, data collection, and analysis. The signal was low-pass filtered at 2,000 Hz. Event time stamps from Med Associates were recorded in order to analyze behaviorally relevant changes in DA release.

DA was identified by its stereotypical and specific cyclic voltammogram signature. Behaviorally evoked DA signals met electrochemical criterion if the cyclic voltammogram was highly correlated with that of the DA templates produced during training sets, as described below. The training set is a template extracted from individual animals that contains six each of background-subtracted cyclic voltammograms and corresponding calibrated concentrations for both DA and pH acquired during electrical stimulations that are known to evoke DA release (stimulation at 1 V: 30 Hz, 6 pulses; 30 Hz, 12 pulses; 30 Hz, 24 pulses; 60 Hz, 6 pulses; 60 Hz, 12 pulses; 60 Hz, 24 pulses). The data collected during a session were not analyzed if DA release did not satisfy these chemical verification criteria (e.g., Fig 1D and 1E). Voltammetric data were analyzed using software written in LabView and Matlab. A principal component regression (Tar Heel CV chemometrics software) was used to extract the DA component from the raw voltammetric data [33,34]. Eigenvalues (principal components) are calculated that describe relevant components of the training set, and we perform multivariate regression analysis to determine a correlation coefficient to describe our recorded behavioral data versus the training set. The number of factors we select to keep in our PCA analysis accounts for >99% of the variance (at least three, but usually four to five factors are kept). Factor selection is a very important step, as retaining more factors than we need would add noise to our data, but retaining too few could mean discarding potentially meaningful information [35]. Importantly, the exact same method was applied to both groups, allowing for fair comparisons.

We also use the residual to examine the quality of the fit. In general, the residual is the difference between the experimental observation and the predicted value derived from a model/template (our regression values) and is a measure of the unknown portion of the signal, which is not accounted for by the principal components of the regression. This is important when considering the accuracy and the applicability of the model and is important for identifying possible interfering molecules or noise (such as drift). The sum of squares of the difference between the template and the experimental data is the residual value (Q) and the threshold, Qa, establishes whether the retained principal components provide a satisfactory description of the experimental data; the discarded principal components should provide a measure of noise [33–35]. We use this Qa measure in combination with our regression analysis to establish our concentration corrections.

Chemometrics is a widely used analytical method that separates changes in current that are caused by DA release from those caused by pH shift or other electrochemical “noise” by comparing eigenvalues derived from stimulated DA release and changes in pH with those derived from behavioral release [4,33–38]. Once converted to concentrations, DA release was examined over three analysis epochs: (1) Baseline = 3 s before CS onset, (2) CS epoch = 3 s starting 1 s after CS onset, and (3) US epoch = 3 s starting 1 s after reward delivery (i.e., lever in). In our final data set, there were 12 rats with 10 sessions per rat for the 120-s ITI group. In the 60-s ITI group, there were 5 rats with 10 sessions, 1 rat with 6 sessions (days 4–9), and 1 rat with 3 sessions (days 4–6).

### Histology

Following the completion of the study, animals were terminally anesthetized with an overdose of isoflurane (5%) and transcardially perfused with saline and 4% paraformaldehyde. Brain tissue was removed and postfixed with paraformaldehyde. Brains were then placed in 30% sucrose solution for 72 h and sectioned coronally (50 μm) using a microtome. Tissue slices were mounted onto slides and stained with thionin for histological reconstruction.

**Disclaimer:** The opinions expressed in this article are the authors’ own and do not reflect the view of the NIH/DHHS.

## Supporting information

S1 Fig Task and electrode placement. **(A)** DA release was recorded during a standard Pavlovian conditioned approach behavior task for 10 d. Each behavioral session consisted of 25 trials presented at a random time interval of either 60 s (± 30; *n* = 7 rats) or 120 s (± 30; *n* = 12 rats). (**B-C**) Placement of chronic recording electrodes within the NAc core [39] based on histology for the 120-s (B) and 60-s (C) groups [39].(**D-E**) False color plots indicate voltammetric current (z-axis) plotted against applied scan potential (y-axis) and time (x-axis), for example, 120-s (D) and 60-s (E) ITI trials. DA, dopamine; ITI, intertrial interval; NAc, nucleus accumbens core.
(TIF)

S2 Fig Sign tracking is more prominent in rats that performed sessions with 120-s ITIs. **(A)** Average beam break (solid) and lever press (dashed) rate for 120-s (red) and 60-s (blue) ITI groups. **(B)** Average lever press rate for 120-s (red) and 60-s (blue) ITI groups. Data are the same as in “A” but with a smaller scale so that differences and timing can be better visualized. Error bars represent SEM, with “*n*” being rat. **(C)** Green lines are the difference between solid blue and red lines from “A” (food cup entries for the 120-s ITI group minus food cup entries for the 60-s ITI group) during the cue period. Thus, negative deflections illustrate more food cup entries during sessions with a 60-s ITI. Orange lines represent the differences between 120-s ITI group lever pressing and 60-s ITI group lever pressing (i.e., red dashed minus blue dashed from “B”). Thus, positive deflections represent time during the cue period when rats in the 120-s ITI group lever pressed more than those in the 60-s ITI group. Orange and green tick marks represent 500-ms bins in which there was a significant difference between 120-s and 60-s ITI groups (*t* test; *p* < 0.05). The way the data are presented here is identical to that in Fig 1G–1I, except data here were averaged within session and rat, and then averaged across rat (120-s ITI, *n* = 12; 60-s ITI, *n* = 7). **(D-F)** Food cup entries (D), lever pressing (E), and the difference between food cup entries and lever pressing (F; lever − food cup) during the first 2 s of the cue period for individual rats. Underlying data for S2 Fig can be found in S4 Data. ITI, intertrial interval.
(TIF)

S3 Fig Development of sign tracking and DA signals over training averaged across rats. **(A-B**) Average beam break (solid) and lever press (dashed) rate for 120-s (A) and 60-s (B) ITI groups. **(C-D)** Average lever press rate for 120-s (C) and 60-s (D) ITI groups. Data are the same as in A and B but with a smaller scale so that differences and timing can be better visualized. **(E-F)** Average DA release over time for 120-s (E) and 60-s (F) ITI rats. In each of the above (A-F), data are broken down into averages from sessions 1–3 (pale colors, pink [120 s] and turquoise [60 s]) and sessions 4–10 (dark colors; red [120 s] and blue [60 s]); 60-s ITI group = 7 rats; 120-s ITI group = 12 rats. Error bars represent SEM, with “*n*” being rat. Data presented here are identical to Fig 3A–3F, except average behavior and DA release within each session and rat were first computed and then averaged across rats. Underlying data for S3 Fig can be found in S5 Data. DA, dopamine; ITI, intertrial interval.
(TIF)

S4 Fig Food cup entries and lever pressing over time for each training session. Lever pressing **(B,D)** and food cup entries **(A,C)** over the trial time for each of the 10 sessions for 120-s ITI **(A,B)** and 60-s ITI **(C,D)** sessions. For the 120-s ITI group, lever pressing was high during the first session and already near maximum levels for that group by the second session (S4B Fig). For the 60-s ITI group, lever pressing was present in the first session and gradually increased through sessions 1–10 (S4D Fig). Thus, it appears that the 120-s ITI group learned to lever press faster, possibly indicating that they learned the relationship between the CS and US earlier. However, if one examines food cup entries during the cue period, it appears that the 60-s ITI also understood the relationship between the CS and US during the very first session. For the 60-s ITI group, food cup entries were higher than 120-s ITI group, starting immediately at the time that the lever (CS) was extended (S4C Fig), and remained high throughout the cue period. Interestingly, for the 60-s ITI group, food cup entries became more fine-tuned, only being present early (during the first 2 s) in the cue period during later sessions. As for the 120-s ITI group, food cup entries during the cue period were modest during the first session and barely present during the second session, during which rats lever pressed at high rates (S4A Fig). These temporal trial dynamics that change over learning will be incorporated into future versions of the model. Underlying data for S4 Fig can be found in S6 Data. CS, conditioned stimulus; ITI, intertrial interval; US, unconditioned stimulus.
(TIF)

S1 Data Underlying data for Fig 1. (XLSX)

S2 Data Underlying data for Fig 2. (XLSX)

S3 Data Underlying data for Fig 3. (XLSX)

S4 Data Underlying data for S2 Fig. (XLSX)

S5 Data Underlying data for S3 Fig. (XLSX)

S6 Data Underlying data for S4 Fig. (XLSX)

## Abbreviations

CS: conditioned stimulus
DA: dopamine
FSCV: fast-scan cyclic voltammetry
GT: goal tracking
GTs: goal trackers
ITI: intertrial interval
MB: model-based
MF: model-free
NAc: nucleus accumbens core
P: Probability
PCA: Pavlovian Conditioned Approach
RL: reinforcement learning
RPE: reward prediction error
ST: sign tracking
STs: sign trackers
STGT: sign tracking and goal tracking
US: unconditioned stimulus
