# Supplementary figures and images for: Manipulating the revision of reward value during the intertrial interval increases sign tracking and dopamine release
Source: PLoS Biol. 2018 Sep 26;16(9):e2004015. doi: 10.1371/journal.pbio.2004015 (PMC6175531; doi:10.1371/journal.pbio.2004015)

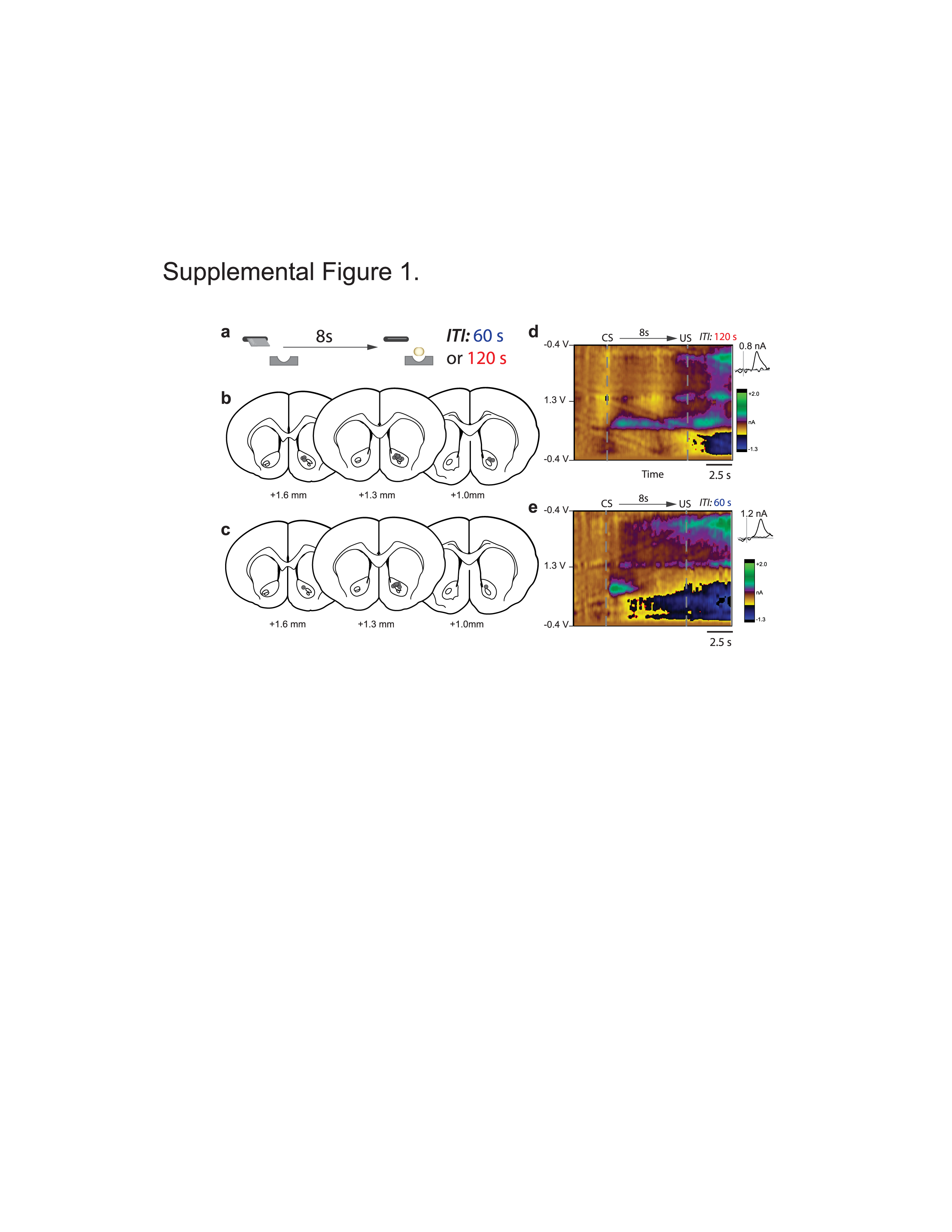

Supplement: S1 Fig — (A) DA release was recorded during a standard Pavlovian conditioned approach behavior task for 10 d. Each behavioral session consisted of 25 trials presented at a random time interval of either 60 s (± 30; n = 7 rats) or 120 s (± 30; n = 12 rats). (B-C) Placement of chronic recording electrodes within the NAc core [39] based on histology for the 120-s (B) and 60-s (C) groups [39].(D-E) False color plots indicate voltammetric current (z-axis) plotted against applied scan potential (y-axis) and time (x-axis), for example, 120-s (D) and 60-s (E) ITI trials. DA, dopamine; ITI, intertrial interval; NAc, nucleus accumbens core. (TIF) [file pbio.2004015.s001.tif]

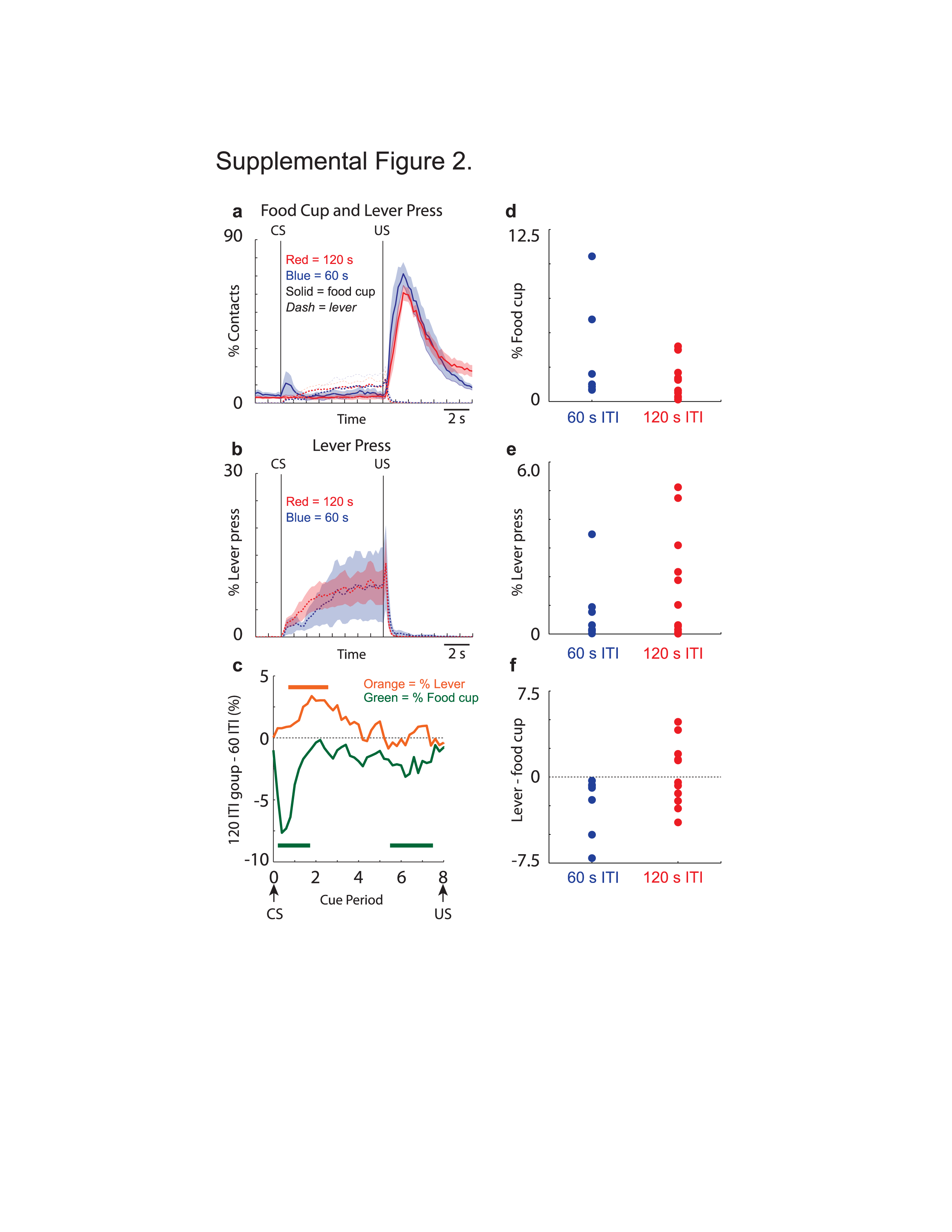

Supplement: S2 Fig — (A) Average beam break (solid) and lever press (dashed) rate for 120-s (red) and 60-s (blue) ITI groups. (B) Average lever press rate for 120-s (red) and 60-s (blue) ITI groups. Data are the same as in “A” but with a smaller scale so that differences and timing can be better visualized. Error bars represent SEM, with “n” being rat. (C) Green lines are the difference between solid blue and red lines from “A” (food cup entries for the 120-s ITI group minus food cup entries for the 60-s ITI group) during the cue period. Thus, negative deflections illustrate more food cup entries during sessions with a 60-s ITI. Orange lines represent the differences between 120-s ITI group lever pressing and 60-s ITI group lever pressing (i.e., red dashed minus blue dashed from “B”). Thus, positive deflections represent time during the cue period when rats in the 120-s ITI group lever pressed more than those in the 60-s ITI group. Orange and green tick marks represent 500-ms bins in which there was a significant difference between 120-s and 60-s ITI groups (t test; p < 0.05). The way the data are presented here is identical to that in Fig 1G–1I, except data here were averaged within session and rat, and then averaged across rat (120-s ITI, n = 12; 60-s ITI, n = 7). (D-F) Food cup entries (D), lever pressing (E), and the difference between food cup entries and lever pressing (F; lever − food cup) during the first 2 s of the cue period for individual rats. Underlying data for S2 Fig can be found in S4 Data. ITI, intertrial interval. (TIF) [file pbio.2004015.s002.tif]

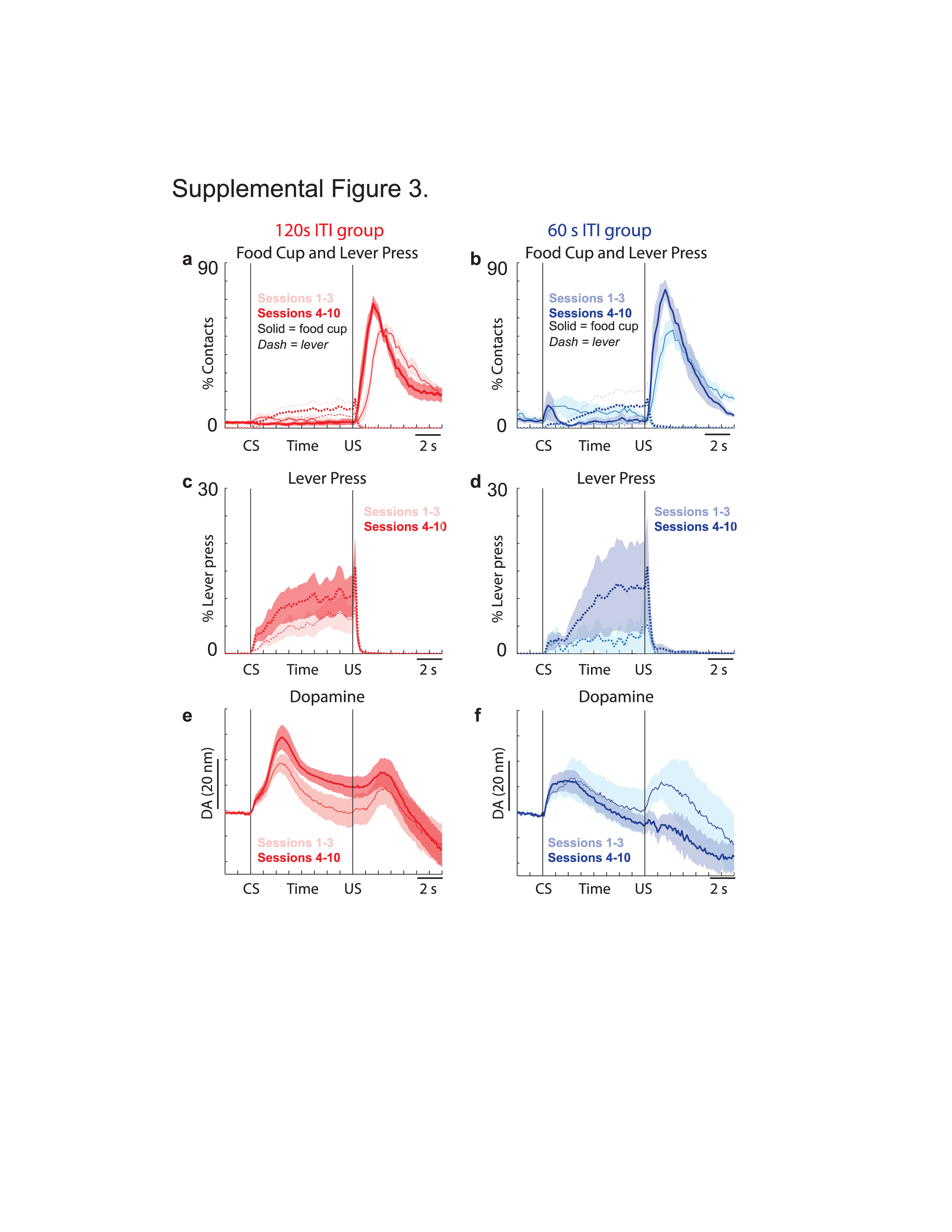

Supplement: S3 Fig — (A-B) Average beam break (solid) and lever press (dashed) rate for 120-s (A) and 60-s (B) ITI groups. (C-D) Average lever press rate for 120-s (C) and 60-s (D) ITI groups. Data are the same as in A and B but with a smaller scale so that differences and timing can be better visualized. (E-F) Average DA release over time for 120-s (E) and 60-s (F) ITI rats. In each of the above (A-F), data are broken down into averages from sessions 1–3 (pale colors, pink [120 s] and turquoise [60 s]) and sessions 4–10 (dark colors; red [120 s] and blue [60 s]); 60-s ITI group = 7 rats; 120-s ITI group = 12 rats. Error bars represent SEM, with “n” being rat. Data presented here are identical to Fig 3A–3F, except average behavior and DA release within each session and rat were first computed and then averaged across rats. Underlying data for S3 Fig can be found in S5 Data. DA, dopamine; ITI, intertrial interval. (TIF) [file pbio.2004015.s003.tif]

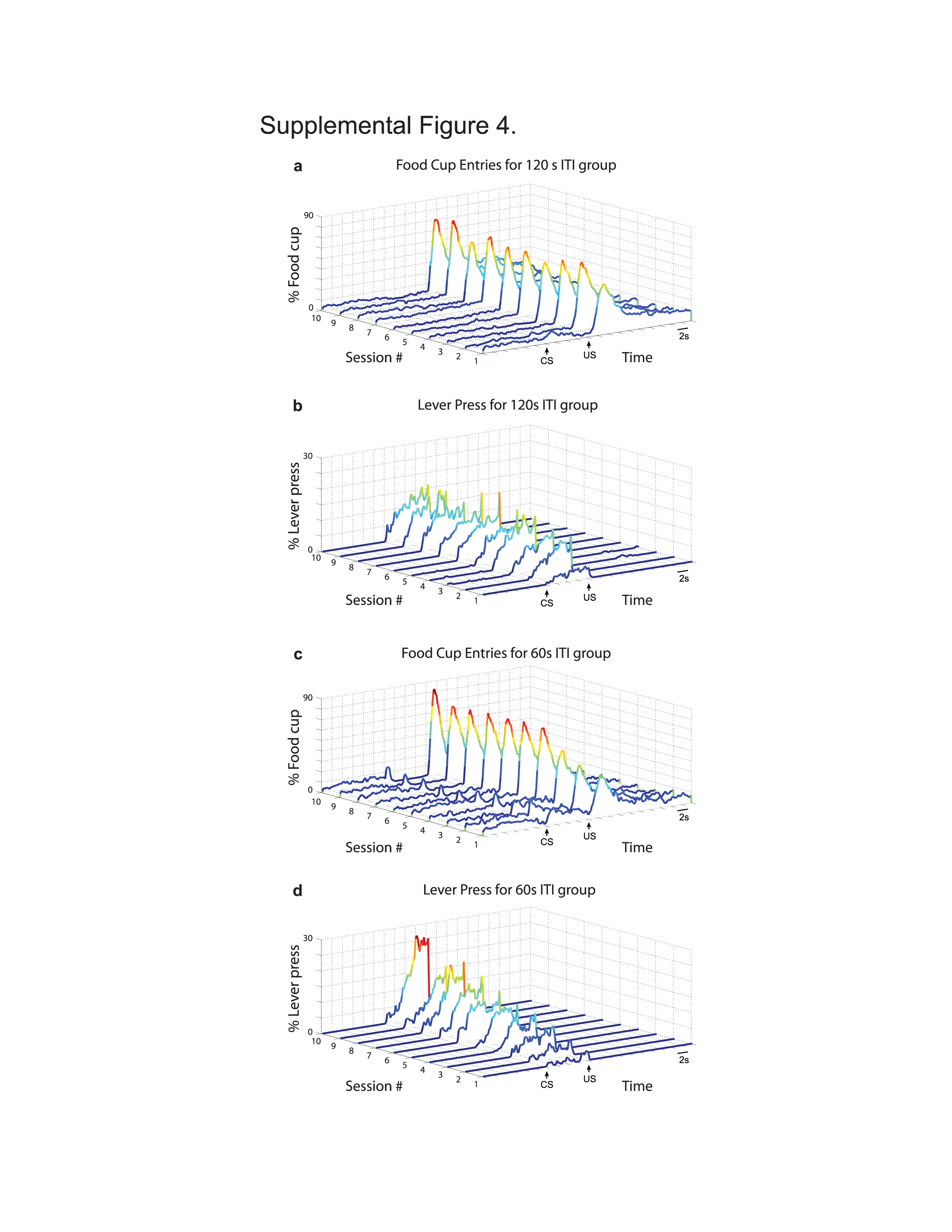

Supplement: S4 Fig — Lever pressing (B,D) and food cup entries (A,C) over the trial time for each of the 10 sessions for 120-s ITI (A,B) and 60-s ITI (C,D) sessions. For the 120-s ITI group, lever pressing was high during the first session and already near maximum levels for that group by the second session (S4B Fig). For the 60-s ITI group, lever pressing was present in the first session and gradually increased through sessions 1–10 (S4D Fig). Thus, it appears that the 120-s ITI group learned to lever press faster, possibly indicating that they learned the relationship between the CS and US earlier. However, if one examines food cup entries during the cue period, it appears that the 60-s ITI also understood the relationship between the CS and US during the very first session. For the 60-s ITI group, food cup entries were higher than 120-s ITI group, starting immediately at the time that the lever (CS) was extended (S4C Fig), and remained high throughout the cue period. Interestingly, for the 60-s ITI group, food cup entries became more fine-tuned, only being present early (during the first 2 s) in the cue period during later sessions. As for the 120-s ITI group, food cup entries during the cue period were modest during the first session and barely present during the second session, during which rats lever pressed at high rates (S4A Fig). These temporal trial dynamics that change over learning will be incorporated into future versions of the model. Underlying data for S4 Fig can be found in S6 Data. CS, conditioned stimulus; ITI, intertrial interval; US, unconditioned stimulus. (TIF) [file pbio.2004015.s004.tif]
